# The Transmission Interfaces Contribute Asymmetrically to the Assembly and Activity of Human P-glycoprotein*

**DOI:** 10.1074/jbc.M115.652602

**Published:** 2015-05-18

**Authors:** Tip W. Loo, David M. Clarke

**Affiliations:** From the Departments of Medicine and Biochemistry, University of Toronto, Toronto, Ontario M5S 1A8, Canada

**Keywords:** ABC transporter, membrane enzyme, membrane protein, protein cross-linking, protein folding

## Abstract

P-glycoprotein (P-gp; ABCB1) is an ABC drug pump that protects us from toxic compounds. It is clinically important because it confers multidrug resistance. The homologous halves of P-gp each contain a transmembrane (TM) domain (TMD) with 6 TM segments followed by a nucleotide-binding domain (NBD). The drug- and ATP-binding sites reside at the interface between the TMDs and NBDs, respectively. Each NBD is connected to the TMDs by a transmission interface involving a pair of intracellular loops (ICLs) that form ball-and-socket joints. P-gp is different from CFTR (ABCC7) in that deleting NBD2 causes misprocessing of only P-gp. Therefore, NBD2 might be critical for stabilizing ICLs 2 and 3 that form a tetrahelix bundle at the NBD2 interface. Here we report that the NBD1 and NBD2 transmission interfaces in P-gp are asymmetric. Point mutations to 25 of 60 ICL2/ICL3 residues at the NBD2 transmission interface severely reduced P-gp assembly while changes to the equivalent residues in ICL1/ICL4 at the NBD1 interface had little effect. The hydrophobic nature at the transmission interfaces was also different. Mutation of Phe-1086 or Tyr-1087 to arginine at the NBD2 socket blocked activity or assembly while the equivalent mutations at the NBD1 socket had only modest effects. The results suggest that the NBD transmission interfaces are asymmetric. In contrast to the ICL2/3-NBD2 interface, the ICL1/4-NBD1 transmission interface is more hydrophilic and insensitive to mutations. Therefore the ICL2/3-NBD2 transmission interface forms a precise hydrophobic connection that acts as a linchpin for assembly and trafficking of P-gp.

## Teaser

**Background:** The P-gp drug-binding domain is linked to the two nucleotide-binding domains (NBDs) by NBD1 and NBD2 transmission interfaces.

**Results:** Only mutations at the NBD2 transmission interface blocked P-gp assembly. Tariquidar repaired all 25 mutants.

**Conclusion:** The mechanism of P-gp folding requires precise hydrophobic interactions at the NBD2 interface.

**Significance:** We identified a linchpin for assembly and repair of ABC proteins.

## Introduction

The ATP-binding cassette (ABC)^2^ family is the largest class of transmembrane (TM) proteins (1). They use ATP hydrolysis to translocate a wide variety of substrates including metabolic products, lipids, peptides, sterols, ions, and drugs across extra- and intracellular membranes.

Human ABC proteins have been the subjects of intense research efforts since most are clinically important. Cystic fibrosis (CF), gout, intrahepatic cholestasis types 2 and 3 (liver bile diseases), Tangier disease (HDL deficiency), Dubin-Johnson syndrome (jaundice), hyperinsulimic hypoglycemia of infancy, pseudoxanthoma elasticum, multidrug resistance, secretory diarrheas, anemia, diabetes, and atherosclerosis are examples of potentially lethal or debilitating conditions caused by genetic mutations or altered activity of one of the 48 human ABC proteins (2).

The most common genetic defect is expression of an ABC processing mutant that is defective in folding and trafficking. The classic example is the ΔF508-CFTR (ABCC7) mutant in CF (3). Other examples includes ABCG2 (gout), ABCB4/ABCB11 (progressive familial intrahepatic cholestasis), ABCA1 (Tangier disease), ABCC2 (Dubin-Johnson syndrome), ABCC6 (pseudoxanthoma elasticum), and ABCC8 (hyperinsulimic hypoglycemia of infancy) (4). A high priority is to understand how processing mutations impact synthesis of ABC proteins and use this knowledge to develop therapies to repair the defects.

Human ABC proteins appear to be particularly sensitive to point mutations because they are multi-domain membrane proteins (core structure of two nucleotide-binding domains (NBDs) and two transmembrane domains (TMDs)) that require formation of specific domain-domain contacts to adopt a native structure (5). ABC proteins differ from other multi-domain proteins because much of the folding occurs post-translationally (6–8).

The human P-glycoprotein (P-gp) drug pump has been a very useful model system for studying repair of ABC protein defective in processing/folding because processing mutations throughout the molecule can be repaired by expression in the presence of drug substrates (9) or by introduction of arginine suppressor mutations into TM segments (10, 11). P-gp is a classic ABC protein as it is a single polypeptide of 1280 amino acids that forms a structure containing two NBDs and two TMDs. Its physiological role is block entry or export toxic compounds out of the body.

The interfaces between the domains play critical roles in the P-gp drug efflux mechanism. Drug substrates bind within a cavity located at the interface between the TMDs (11–15). Two ATP-binding sites are located at the interface between the NBDs. ATP hydrolysis occurs by an alternating site mechanism (16–20).

Coupling of ATP hydrolysis in the NBDs to drug efflux from the drug-binding sites in the TMDs is mediated by ball-and-socket joints (21) at the NBD-TMD transmission interfaces. Ball-and-socket joints are intracellular loops (ICLs) connecting TM segments that contain a central intracellular helix (IH) that is in close contact to an NBD. Contacts between TMD1-NBD1 or TMD2-NBD2 are mediated by the first ICL of each TMD (ICL1 or ICL3). The second ICL of each TMD mediates TMD1-NBD2 (ICL2) or TMD2-NBD1 (ICL4) contacts.

Contacts between the homologous halves (TMD1-NBD2 or TMD2-NBD1) appear to be particularly important for mammalian drug pumps. For example, the BCRP/ABCG2 drug pump lacks contacts equivalent to P-gp ICL1/ICL3 (22). In addition, we found that mutations to IH1 (ICL1) had little effect on activity or maturation while mutations to IH2 (ICL2) suggested the presence of a hydrophobic interface that was highly sensitive to changes (23). The presence of such an interface was unexpected since modeling studies suggested that charged residues would play a dominant role (24).

Hypersensitivity to mutation at the IH2/NBD2 transmission interface compared with the IH1/NBD1 interface might explain the differences in the maturation of NBD2 deletion mutants of P-gp and CFTR (25). Deletion of NBD2 inhibits folding and trafficking of P-gp but not CFTR although both are structurally similar ABC proteins (26). It is possible that ICL2/ICL3 interactions with NBD2 are critical for P-gp maturation and the protein is destabilized when NBD2 is deleted. Understanding P-gp domain-domain interactions will also be of importance when extrapolated to folding defects of other mutant ABC proteins associated with disease and in the design of methods to counteract their effects. It has been shown that in processing mutations of P-gp or CFTR trap the proteins as partially folded intermediates with incomplete NBD-TMD interactions (7).

In this study, we tested whether the NBD2 transmission interface was particularly critical for P-gp assembly and activity by comparing the effects of mutations at the NBD1-TMD and NBD2-TMD interfaces. We report that the interfaces are asymmetric since P-gp maturation was only sensitive to mutations at the NBD2 interface. The results suggest that the NBD2-TMD interface is a linchpin for assembly and repair of ABC proteins.

## Experimental Procedures

### 

#### 

##### Construction of Mutants

Mutations were introduced into the wild-type or Cys-less P-gp cDNAs containing the A52-epitope or 10-histidine tags (27) by site-directed mutagenesis as described by Kunkel (28). In most cases, residues in ICL1 (residues 146–177), ICL2 (residues 250–277), ICL3 (residues 787–818) or ICL4 (residues 893–920) were replaced with alanines to test for effects on maturation. Exceptions were that alanines were replaced with leucine, glycines were replaced with valine, and leucines or valines were replaced with serine as performed previously (23, 29, 30). Mutants were constructed to contain an A52 epitope tag at their C-terminal ends for use in whole cell immunoblot assays (29). The presence of the epitope tag distinguished the mutant proteins from any endogenous P-gp. P-gp contains three *N*-linked glycosylation sites can be used to monitor maturation of human P-gp from an immature 150 kDa protein to a mature 170 kDa protein. P-gp cDNA was modified to contain a 10-histidine tag at the COOH-terminal end to facilitate purification of the expressed protein by nickel-chelate chromatography (31).

##### Expression and Maturation of ICL Mutants

The ICL mutant cDNAs were transiently expressed in HEK 293 cells by a calcium phosphate precipitation approach as described previously (7). Briefly, 10 μl of 2.5 m CaCl_2_ was added to 90 μl H_2_O containing 2 μg of DNA followed by addition of 100 μl of BES solution (50 mm N,N-bis(2-hydroxyethyl)-2-aminoethanesulfonic acid, 280 mm NaCl and 1.5 mm Na_2_HPO_4_, pH 6.96). After 10 min at room temperature, 4 ml of HEK 293 cells (about 100,000 cells/ml) in Dulbecco's modified Eagle's medium (DMEM) with high glucose (supplemented with nonessential amino acids, 4 mm l-glutamine, 10 IU/ml penicillin, 10 μg/ml streptomycin, and 10% (*v*/*v*) bovine calf serum) was added and 1.5 ml of the mixtures were added to duplicate well of 6-well culture plates. After 5 h at 37 °C, the medium was replaced with fresh medium with or without 0.5 μm tariquidar. About 16 h later, the cells were harvested, washed with PBS, and cell pellets suspended in 150 μl of 2× SDS sample buffer (125 mm Tris-HCl, pH 6.8, 4% (*w*/*v*) SDS, 4% (*v*/*v*) 2-mercaptoethanol containing 25 mm EDTA. Samples were applied to 6.5% SDS-PAGE gels (minigels, 1.5 mm spacers, 15 wells). The gels were electroblotted onto a sheet of nitrocellulose and P-gp proteins detected using A52 monoclonal antibody, horseradish peroxidase conjugated anti-mouse secondary antibody, and enhanced chemiluminescence. The signals were imaged and levels of mature (170 kDa) P-gp relative to total P-gp (mature 170 kDa plus immature 150 kDa) determined using Chemidoc^TM^ XRS^+^ with Image Lab^TM^ software (Bio-Rad Lab. Inc., Mississauga, Ontario). An equivalent amount of the sample was loaded onto 10% (*v*/*v*) SDS-PAGE gels and subjected to immunoblot analysis with a monoclonal antibody against glyceraldehyde-3-phosphate dehydrogenase (GADPH) (internal control).

##### Purification of P-gp and Measurement of ATPase Activity

HEK 293 cells were plated onto fifty plates (10-cm diameter) and transfected with the cDNA of the histidine-tagged P-gp mutant when about 50% confluent. After 16 h at 37 °C, the medium was replaced with fresh medium containing 5 μm cyclosporin A. Cyclosporin A is a substrate of P-gp and acts as a potent pharmacological chaperone in promoting maturation and yield of P-gp (9). Cyclosporin A rather than tariquidar was used to rescue the mutants for purification because it is considerably less expensive when used in scaled up experiments. The cells were harvested after another 24 h at 37 °C and washed three times with phosphate-buffered saline (PBS, pH 7.4). The cells were then suspended in PBS and solubilized at 4 °C by addition of one volume (0.75 ml) of PBS containing 2% (w/v) *n*-dodecyl-β-d-maltoside (Anatrace Inc., Maumee, OH). After 10 min at 4 °C, insoluble material was removed by centrifugation at 16 000 × *g* for 15 min at 4 °C. DNA in the supernatant was removed by passage through a DNA miniprep microfuge column (Bio Basic Canada Inc., Markham, ON). The flow-through material was then applied onto a nickel spin column (Ni-NTA, Qiagen, Mississauga, ON) that had been pre-equilibrated with buffer A containing 50 mm NaPO_4_, pH 8.0, 500 mm NaCl, 50 mm imidazole and 20% (v/v) glycerol and 0.1% (w/v) n-dodecyl-β-d-maltoside. The column was then washed twice with 0.6 ml buffer B containing 10 mm Tris-HCl, pH 7.5, 500 mm NaCl, 80 mm imidazole, pH 7.0, 20% (*v*/*v*) glycerol and 0.1% (*w*/*v*) n-dodecyl-β-d-maltoside and twice with 0.6 ml buffer C (buffer B containing 50 mm imidazole). Histidine-tagged P-gp was then eluted with 0.2 ml of buffer B but containing 300 mm imidazole. Recovery of P-gp was monitored by immunoblot analysis with rabbit anti-P-gp polyclonal antibody (32). A sample of the isolated histidine-tagged P-gp (about 100 ng) was mixed with an equal volume of 10 mg/ml sheep brain phosphatidylethanolamine (Type II-S, Sigma) that had been washed and suspended in TBS. ATPase activity (33) was measured in the presence of 0.4 mm verapamil.

##### Disulfide Cross-linking Analysis

Cys-less P-gp or the ICL1/ICL4 double cysteine mutants I160C/F904C or F163C/R905C were transiently expressed in HEK 293 cells at reduced temperature (30 °C) to promote maturation. The P-gps were isolated by nickel-chelate chromatography. The isolated P-gps were incubated 20 °C for 10 min in the presence or absence of 0.5 mm copper phenanthroline (oxidant to promote disulfide bond formation). EDTA was then added to a final concentration of 2 mm. Immunoblot analysis and assay of ATPase activity were performed as described above.

## Results

### 

#### 

##### Maturation of P-gp is Highly Sensitive to Mutations at the NBD2-TMD Interface (ICL2/3) but Insensitive to Mutations at the NBD1-TMD (ICL1/4) Interface

**FIGURE 1.**
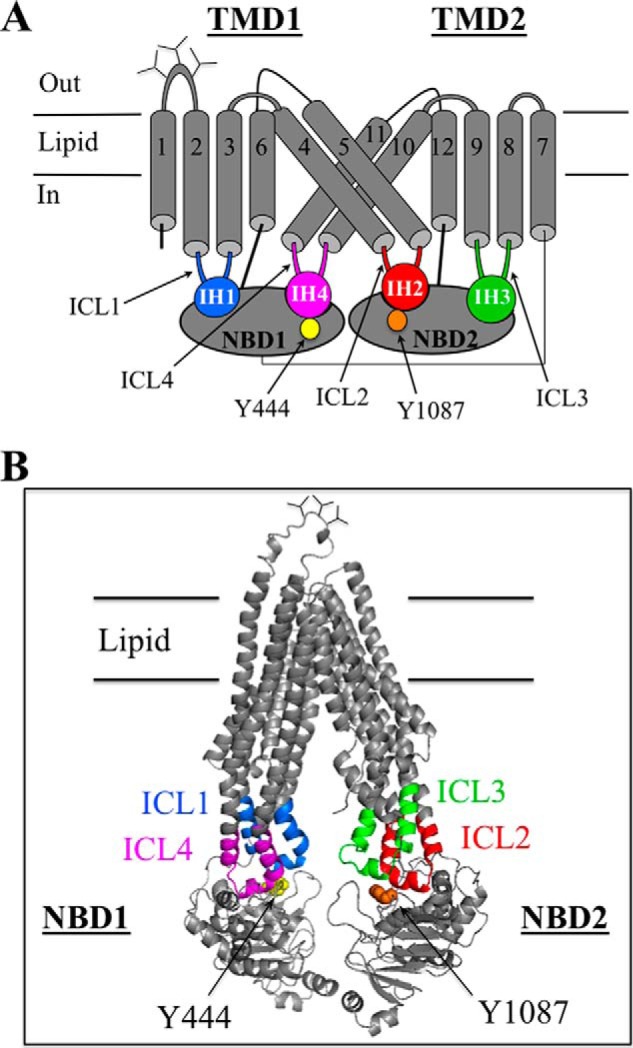
**Models of human P-gp.** *A*, secondary structure of human P-gp showing the four ball-and-socket joints of the NBD-TMD transmission interfaces. The intracellular loops (*ICL*s) containing intracellular helices (*IH*s) that interact with the NBDs are shown in color. The cylinders represent TM segments, and the branched lines in the loops connecting TM segments 1 and 2 represent glycosylated sites. *B*, predicted structure of human P-gp in an open conformation was based on the crystal structure of mouse P-gp (56). The intracellular loops interacting with the NBDs are colored. The model was viewed using the PyMol system (57).

The NBDs of P-gp are linked to the TMDs by four ICLs (Fig. 1, *A* and *B*). The IH located in the middle of the ICLs interacts with the NBDs to form ball-and-socket joints (21). The NBD1 transmission interface is linked to TMD1 and TMD2 by ICL1 and ICL4, respectively. The NBD2 transmission interface is linked to TMD1 and TMD2 by ICL2 and ICL3, respectively.

The transmission interfaces might play critical roles to promote folding of P-gp into a native structure. P-gp is initially synthesized in the endoplasmic reticulum to yield a protease-sensitive loosely folded protein with incomplete packing of the TM segments and incomplete domain-domain interactions (34–36). P-gp then matures into a compact protease-resistant native conformation that leaves the endoplasmic reticulum for addition of complex carbohydrate in the Golgi and trafficking to the plasma membrane (36). Removal of the NBDs inhibits maturation of P-gp. P-gp is different from CFTR because deletion of NBD2 only inhibits P-gp maturation (26). This indicated that the NBD2-TMD interface might play a particularly important role in the mechanism of P-gp folding. Truncation mutants lacking NBD2 or both NBDs are trapped in the endoplasmic reticulum in protease-sensitive loosely folded conformations (37).

A mutational approach was used to test if residues in the ICLs at the NBD2 transmission interface played more important roles in maturation of P-gp compared with residues in the ICLs at the NBD1 interface. Maturation of P-gp can readily be monitored in whole cell assays as the protein contains three N-glycosylation sites in the extracellular loop connecting TM segments 1 and 2 (Fig. 1*A*). The protein is initially synthesized as a 150 kDa core-glycosylated protein. If the protein correctly folds into a compact structure it can exit the endoplasmic reticulum for modification of the carbohydrate in the Golgi to yield a 170 kDa mature protein.

Accordingly, 84 mutants were constructed that contained point mutations to residues in IH4/ICL4 and residues in ICL1, ICL2, and ICL3 flanking IH1, IH2, and IH3, respectively (segments of about 30 residues). The 36 IH1, IH2, and IH3 mutants constructed in previous studies (23, 38) were included for comparison.

Mutations were made to amino acids flanking each IH as it had been reported that some of these residues contributed to NBD-TMD interactions in the crystal structure of P-gp from *Caenorhabditis elegans* (21). In general, residues were replaced with alanine as it has a small side chain. Exceptions were that alanines were replaced with leucine, glycines were replaced with valine, and leucines or valines were replaced with serine as performed previously (23, 29, 30).

**FIGURE 2.**
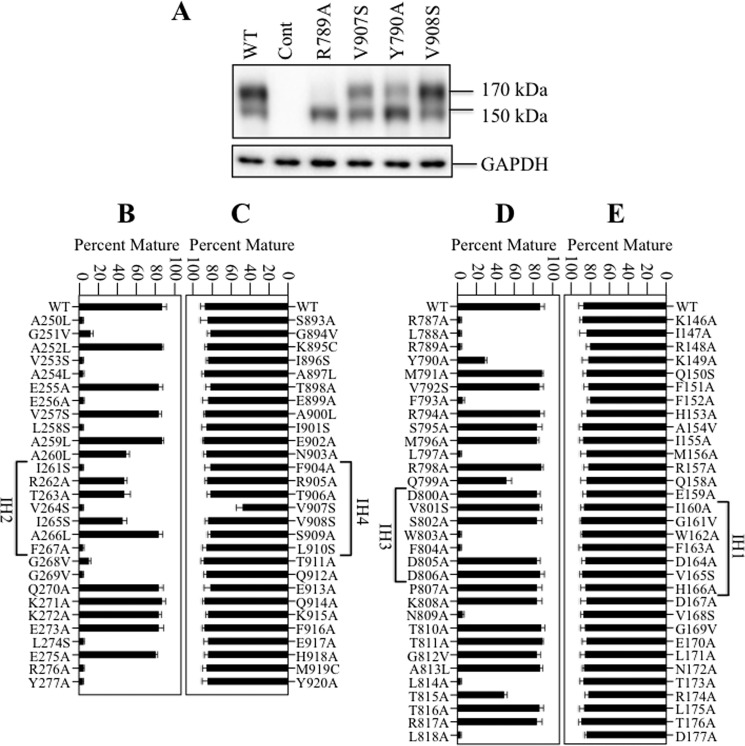
**Maturation of P-gp is highly sensitive to point mutations at the NBD2 transmission interface (ICL2/ICL3) but not point mutations at the NBD1 transmission interface (ICL1/ICL4).** HEK 293 cells were transiently transfected with A-52-tagged P-gps containing point mutations in the ICLs. *A*, representative immunoblot of SDS extracts of whole cells transfected with wild-type P-gp (WT), control vector (Cont), or P-gp mutants R789A, V907S, Y790A, or V908S showing no maturation (R789A), partial maturation (V907S, Y790A) or maturation similar to wild-type P-gp (V908S). The positions of mature (170 kDa) and immature (150 kDa) forms of P-gp are indicated. The amount of mature 170 kDa P-gp relative to total (mature 170 kDa plus immature 150 kDa protein) was determined for wild-type P-gp (WT) or mutants with point mutations in ICL2 (*B*), ICL4 (*C*), ICL3 (*D*), or ICL1 (*E*) was determined (Percent Mature). Each value is the mean ± S.D. Residues in the predicted IH segments are indicated.

Mutants were transiently expressed in HEK 293 cells for about 16 h and whole cell SDS extracts were subjected to immunoblot analysis to determine the steady state levels of mature and immature forms of P-gp. Examples of the effects of the mutations compared with wild-type P-gp are shown in Fig. 2*A*. Wild-type P-gp showed efficient maturation as about 80% of the protein was present as the 170 kDa mature form of the protein. The remainder of the protein was the 150 kDa immature P-gp. We previously showed that the 150 kDa protein was core-glycosylated as it was sensitive to endoglycosidases H and F (39). The 170 kDa P-gp was sensitive only to endoglycosidase F.

Mutant R789A (ICL3) represents an example of a mutation that blocked maturation as only the 150 kDa immature form of P-gp was detected (Fig. 2*A*). Mutants V907S (IH4) and Y790A (ICL3) represent examples of mutations that partially inhibited maturation. The V907S mutant yielded about equivalent levels of mature and immature P-gp. Mutation of Tyr-790 to alanine substantially reduced maturation as about 85% of the product was the 150 kDa immature protein. Mutant V908S (IH4) represents an example of a mutation that did not detectably reduce P-gp maturation. In general, the mutations did not markedly reduce the steady-state levels of P-gp expression 16 h after transfection. There was less than a 2-fold change in total P-gp compared with wild-type P-gp. For example, the total P-gp yields in mutants R789A, V907S, Y790A, and V908S (Fig. 2*A*) were about 60, 75, 75, and 98% of wild-type P-gp, respectively.

The effects of ICL mutations are shown in Fig. 2 (*panels B–E*). Maturation of P-gp was quite insensitive to mutations at the NBD1 transmission interface. None of the 32 residues in ICL1 when mutated affected maturation of P-gp (Fig. 2*E*; >80% mature P-gp). Similarly, the mutations to residues in ICL4 (except for V907S) also did not affect maturation of P-gp. Mutant V907S yielded a slightly lower amount (about 55%) of mature P-gp.

By contrast, the NBD2 transmission interface was highly sensitive to point mutations (Fig. 2, *B* and *D*). Fourteen of the twenty-eight mutations in ICL2 (A250L, G251V, V253S, A254L, E256A, L258S, I261S, V264S, F267A, G268V, G269V, L274S, R276A, Y277A) inhibited maturation of P-gp (<15% mature P-gp) while four other ICL2 mutations (A2650L, R262A, T263A, and I265S) partially reduced maturation of P-gp (about 55–60% mature P-gp). The results suggest that the NBD1 and NBD2 transmission interfaces make asymmetric contributions to maturation of P-gp. Maturation of P-gp was highly sensitive to mutations that could disrupt formation of the ICL2/ICL3 tetrahelix bindle at the NBD2 transmission interface.

##### Rescue of Processing Mutants

**FIGURE 3.**
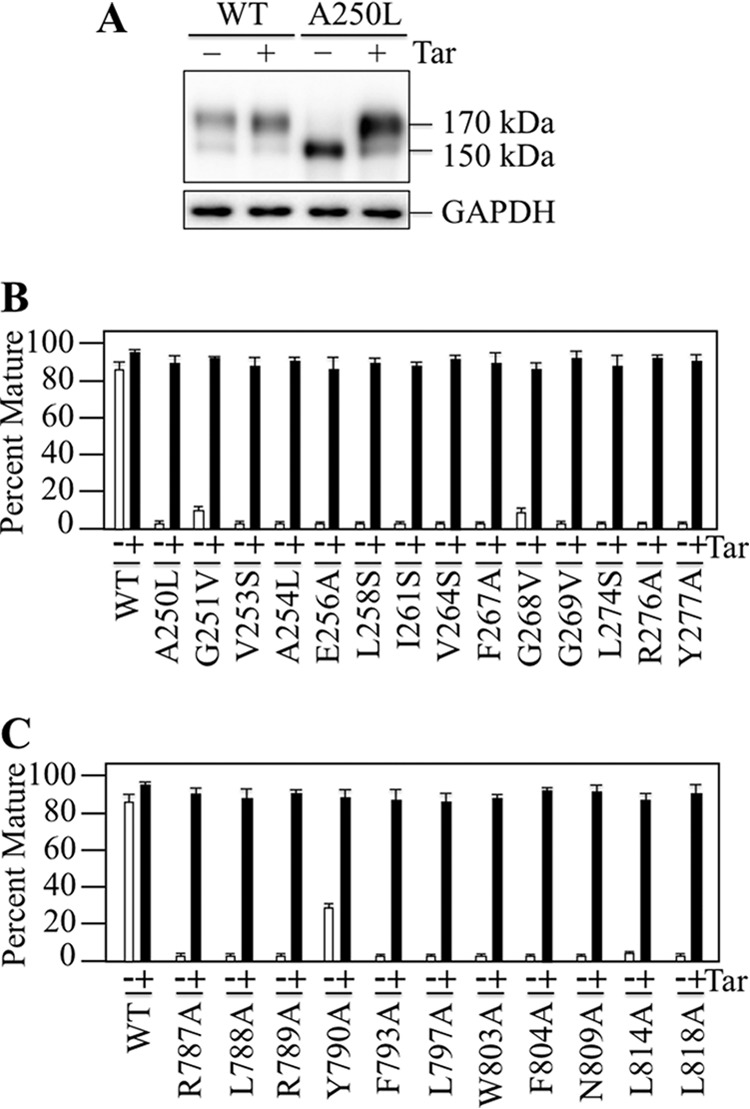
**Rescue of P-gp Processing Mutants with Tariquidar.** HEK 293 cells were transiently transfected with A52-tagged wild-type P-gp or ICL processing mutants that yielded immature P-gp as the major product (Fig. 2) and expressed with (+) or without (−) 0.5 μm tariquidar (Tar). *A*, representative immunoblot of SDS extracts of whole cells transfected with wild-type P-gp (WT) or mutant A250L. The positions of mature (170 kDa) and immature (150 kDa) forms of P-gp are indicated. The amount of mature P-gp relative to total for wild-type P-gp or mutants with processing mutations in ICL2 (*B*) or ICL3 (*C*) was determined (Percent Mature). Each value is the mean ± S.D. (*n* = 3 different transfections).

Drug substrates and modulators can act as pharmacological chaperones to rescue P-gp processing mutants (9, 40). There were 25 ICL mutations at the NBD2 transmission interface that yielded immature 150 kDa P-gp as the major product (Fig. 2, *panels B–E*). To test if maturation of the processing mutants could be restored, they were expressed in the presence of tariquidar. Tariquidar was selected because it is the most potent pharmacological chaperone for rescue of P-gp processing mutants (41). For example, tariquidar but not cyclosporine A could repair the F804D mutation at the ICL3-NBD1 interface (41). Rescue of a representative misprocessed mutant by tariquidar is shown on Fig. 3*A*. No detectable mature P-gp was observed when mutant A250L (ICL2) was expressed in the absence of drug substrate. Expression in the presence of tariquidar however, promoted maturation of the A250L mutant to yield mature 170 kDa P-gp as the major product. Accordingly, all 25 of the ICL processing mutants were expressed in HEK 293 cells in the absence or presence of tariquidar. A sample of whole cell SDS extracts was subjected to SDS-PAGE and immunoblot analysis and the amount of mature 170 kDa P-gp was quantified. It was found that all of the misprocessed mutants could be efficiently rescued with tariquidar to yield mature P-gp as the major product (Fig. 3, *B* and *C*; >90% mature P-gp). The results show that the defects caused by the ICL3 (Fig. 3*B*) or ICL4 (Fig. 3*C*) point mutations at the NBD2 transmission interface could be overcome by binding of tariquidar to the TMDs. We previously showed that a P-gp truncation mutant lacking the NBDs could be efficiently rescued with tariquidar (41).

##### The IH4-NBD1Contact Site Is Less Hydrophobic than the Equivalent IH2-NBD2 Site

Mutational analysis of the ICLs (Fig. 2) suggested that the NBD1 and NBD2 transmission interfaces were asymmetric in their contribution to P-gp folding. To test if these interfaces also contributed asymmetrically to activity, we examined whether the hydrophobic residues at the TMD2(IH4)-NBD1 site were critical for activity because the TMD1(IH2)-NBD2 transmission interface appeared to have a hydrophobic contact site (23). The IH2-NBD2 joint was hydrophobic because replacement of Phe-1086 or Tyr-1087 aromatic residues at the NBD2 socket (Fig. 1) with small or hydrophilic residues inhibited P-gp maturation and activity. For example, the F1086A mutation abolished activity. Activity of the F1086A could be restored if the opposing Ala-266 residue in IH2 was replaced with an aromatic residue (23).

**FIGURE 4.**
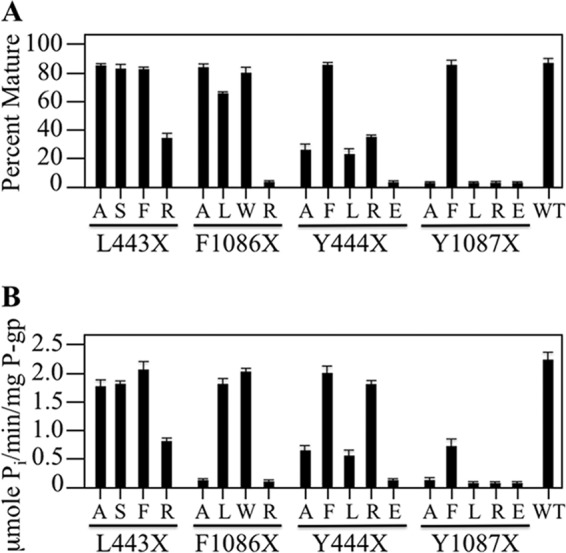
**Point mutations at the NBD2-TMD contact point have a greater impact on P-gp maturation and activity than those at the NBD1-TMD site.** *A*, HEK 293 cells were transiently transfected with A52-tagged wild-type P-gp (WT) or mutants with changes to residues in NBD1 that is adjacent to IH4 (Leu-443, Tyr-444) or to homologous residues in NBD2 (Phe-1086, Tyr-1087). Whole cell SDS extracts were subjected to immunoblot analysis and the amount of mature P-gp relative to total was determined (Percent Mature). Each value is the mean ± S.D. (*n* = 3). *B*, histidine-tagged mutants were expressed in the presence of cyclosporine A to promote maturation. P-gps were isolated, mixed with lipid and ATPase activity determined in the presence of verapamil. The results are derived from three different transfections + S.D. (*n* = 3 different transfections).

Residues equivalent to Phe-1086 and Tyr-1087 at the IH4-NBD1 site are Leu-443 and Tyr-444. To test if the IH4-NBD1 contact point was also hydrophobic we first compared the effects of changes to Leu-443 and Phe-1086 on maturation and activity. Accordingly, A52-tagged mutants L443X (X = A, S, F, or R) were constructed. These mutants and A52-tagged mutants F1086X (X = A, L, W, or R) (23) were transiently expressed in HEK 293 cells and whole cell SDS extracts subjected to immunoblot analysis. The amount of mature P-gp was then quantified. All of the L443X mutations had relatively minor effects on maturation of P-gp (Fig. 4*A*). The amount of mature P-gp in mutants L443A, L443S, and L443F P-gps were similar to wild-type while mutant L443R yielded about 35% mature P-gp. Mutants F1086A, F1086L, and F1086W yielded mature 170 kDa protein as the major product, whereas F1086R yielded little mature P-gp (<5%) (Fig. 4*A*).

Histidine-tagged versions of the L443X (X = A, S, F, or R) and F1086X (X = A, L, W, or R) mutants were expressed in HEK 293 cells in the presence of cyclosporine A to promote maturation of the mutants. Expression in the presence of cyclosporine A promoted maturation of all the mutants to wild-type levels (data not shown). The mutants were isolated and ATPase activity measured in the presence of verapamil. Verapamil was used because it is a substrate (42) that highly activates P-gp ATPase activity (over 10-fold) (43). There is a good correlation between drug transport and activation of ATPase activity (44).

It was found that P-gp was again less sensitive to changes at the Leu-443 position. While the F1086A and F1086R mutations blocked verapamil stimulated ATPase activity, the L443A mutant resembled wild-type activity, and the L443R mutant retained about 35% activity (Fig. 4*B*).

It was possible that the asymmetric effects of changes to positions 443 and 1086 were related to the fact that different amino acids were found at the two locations in the wild-type protein (Leu-443 at NBD1 and Phe-1086 in NBD2). Therefore, we next examined the effects of changes to the residues at the Tyr-444(NBD1) and Tyr-1087(NBD2) positions (Fig. 1).

A52-tagged mutants Y444X were constructed so that tyrosine was replaced with a small (Ala), hydrophobic (Phe, Leu) or charged (Arg, Glu) residue. For comparison, the previously constructed Y1087A, Y1087F, and Y1087L mutants were included (38) and additional mutants Y1087R and Y1087E were also constructed. The mutants were transiently expressed in HEK 293 cells and whole cell SDS extracts subjected to immunoblot analysis. The amount of mature P-gp was quantified. All of the Y1087X mutations except for Y1087F blocked P-gp maturation (Fig. 4*A*). By contrast, the amount of mature P-gp in Y444F and Y1087F was similar to that of wild-type P-gp (Fig. 4*A*). All the Y444X mutants except Y444E differed from their Tyr-1087 counterparts as they yielded detectable levels of mature 170 kDa P-gp. The Y444A and Y444L mutants yielded about 25% mature P-gp while the Y444R mutant yielded about 40% mature P-gp (Fig. 4*A*).

We then tested whether the Y443X and Y1087X mutants had activity. Histidine-tagged mutants were expressed in HEK 293 cells in the presence of cyclosporine A to promote maturation of the mutants. The mutant P-gps were isolated, mixed with lipid and assayed for ATPase activity in the presence of saturating concentrations of verapamil. Fig. 4*B* shows that Y1087F retained about 30% of the activity of wild-type enzyme. The activity of wild-type P-gp was 2.2 + 0.2 μmol P_i_/min/mg protein. The activity of Y1087 was almost completely inhibited when it was mutated to A, L, R, or E. In contrast, the Y444X mutations had less severe effects. With the exception of Y444E, the Tyr-444 mutations had less severe effects on P-gp activity. Both the Y444F and Y444R mutants showed over 75% of wild-type activity. Mutants Y444A and Y444L showed about 25% activity.

Results suggest that the IH4-NBD1 contact site is less sensitive to mutations and less hydrophobic than the equivalent IH2-NBD2 site. The difference is particularly evident when the aromatic residues were replaced with the positively charged arginine residue. Replacement of Phe-1086 or Tyr-1087 with arginines blocks P-gp maturation and activity. The L443R and Y444R mutations only showed modest reductions (Fig. 4). The results show that the NBD1 and NBD2 transmission interfaces contribute asymmetrically to activity.

##### Clamping of IH1 to IH4 Inhibits Activity

Clamping the IH segments in close proximity by cysteine cross-linking can activate or inhibit P-gp ATPase activity. For example, cross-linking of cysteines in ICL1 and ICL3 to hold IH1 and IH3 in close proximity activated P-gp ATPase activity over 10-fold in the absence of drug substrates (45). By contrast, cross-linking of cysteines in IH2 and IH3 together (NBD2 transmission interface) did not activate basal ATPase activity and blocked drug-stimulated ATPase activity (38).

Since IH1 and IH4 at the NBD1 transmission interface were less sensitive to point mutations compared with IH2 and IH3 at the NBD2 transmission interface (Fig. 2), it was possible that P-gp activity might not be inhibited if IH1 were cross-linked to IH4. To test this, the first step was to identify pairs of cysteines in IH1 and IH4 that could be cross-linked. Pairs of cysteines were introduced into the N-terminal segments of IH1 (Glu-159 to Asp-164) and IH4 (Phe-904, Arg-905) of Cys-less P-gp. Cysteines were placed in the N-terminal regions of IH1 and IH4 as this was the strategy employed to identify disulfide cross-linking between IH2 (A259C) and IH3 (W803C). The 12 double cysteine histidine-tagged mutants were expressed in HEK 293 cells at low temperature (30 °C) to promote maturation. The mutants were isolated by nickel-chelate chromatography and subjected to cross-linking and assayed for activity (data not shown).

**FIGURE 5.**
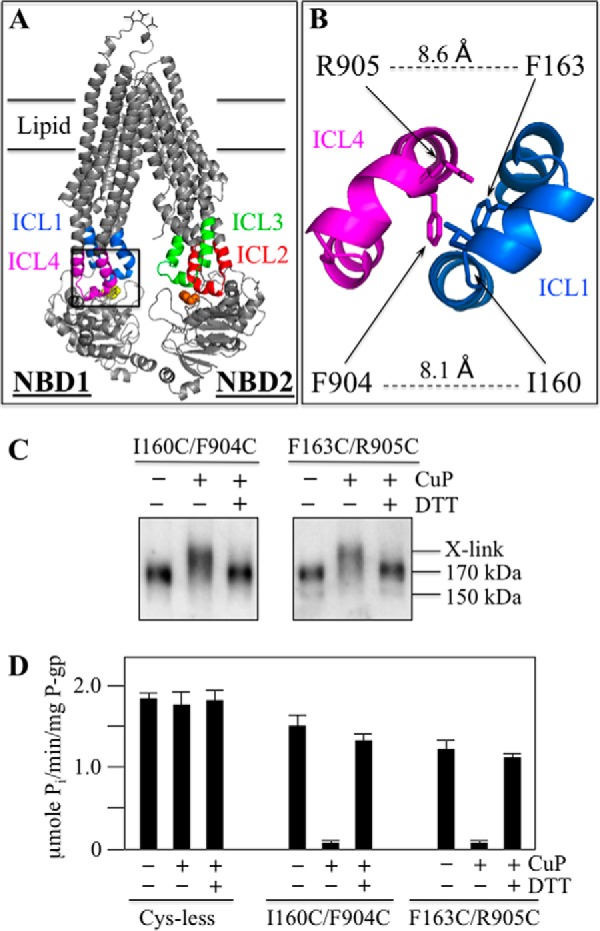
**IH1/IH4 cross-linking inhibits P-gp ATPase activity.** *A*, model of human P-gp showing parts of the various ICLs. The *boxed inset* is expanded in *B* to show the positions of residues I160C(IH1), F904C(IH4), F163C(IH1), and R905C(IH4). The distances (Å) between the α carbons of I160(IH1)/F904(IH4) and F163(IH1)/R905(IH4) are indicated. *C*, histidine-tagged Cys-less P-gp or mutants containing pairs of cysteines introduced into IH1 and IH4 (I160C(IH1)/F904C(IH4), F163C(IH1)/R905C(IH4)) (in Cys-less background) were transiently expressed in HEK 293 cells and isolated by nickel-chelate chromatography. Samples of the isolated P-gps were treated without (−) or with (+) copper phenanthroline (CuP). The reactions were stopped by addition of EDTA. Samples from the mutant P-gps were subjected to immunoblot analysis before (−) or after (+) treatment with dithiothreitol (DTT). The positions of cross-linked (X-link), mature (170 kDa), and immature (150 kDa) P-gps are indicated. *D*, samples were also assayed for ATPase activity in the presence of verapamil. Each value is the mean ± S.D. (*n* = 3).

Mutants I160C/F904C and F163/R905C (Fig. 5, *A* and *B*) were selected to test the effects of IH1/IH4 cross-linking as the mutants yielded mature P-gp that showed efficient cross-linking in the presence of oxidant (copper phenanthroline) (Fig. 5*C*). Cross-linking can readily be detected because cross-linking between different domains causes P-gp to migrate slower on SDS-PAGE gels (7). The cross-linked products disappeared when samples were treated with dithiothreitol prior to immunoblot analysis (Fig. 5*C*). No cross-linked product was observed when the I160C, F904C, F163C, or R905C mutants were treated with oxidant (data not shown).

To test for the effects of cross-linking on activity, isolated Cys-less P-gp and mutants I160C/F904C and F163/R905C were treated without or with copper phenanthroline for 10 min a 20 °C. The reaction was stopped by addition of EDTA. The samples were then assayed for verapamil-stimulated ATPase activity. Before treatment with oxidant, both mutants exhibited robust drug-stimulated ATPase activity. Mutants I160C/F904C and F163C/R905C exhibited over 70% of the activity of the Cys-less parent (Fig. 5*D*). Cross-linking of mutants I160C/F904C and F163C/R905C with oxidant however, inhibited more than 90% of the activity. Treatment with oxidant had little effect on the Cys-less parent. Activity of the cross-linked mutants was restored when the disulfide bond was reduced with dithiothreitol (Fig. 5*D*).

The results show that IH1/IH4 and IH2/IH3 cross-linking had similar effects as they both severely inhibited the activity of P-gp. The results are in agreement with modeling studies that predict that the IH1/IH4 and IH2/IH3 segments undergo significant conformational changes during the reaction cycle that would alter the relative positions of residues at the IH1/IH4 and IH2/IH3 interfaces (24, 46). Although the transmission interfaces contribute asymmetrically to folding and activity, the cross-linking studies show that movement between the helices at both interfaces are important for activity.

##### The R262A(IH2)/R905R(IH4) Mutant Retains Robust ATPase Activity

Pajeva *et al.* (24) predicted that homologous arginines at positions 262 (IH2) and 905 (IH4) were critical for coupling ATP hydrolysis to drug efflux. We found however, that the R905C mutation caused only a modest reduction in drug-stimulated ATPase activity (Fig. 5*D*).

A study of Q loop mutations Q475A(NBD1) and Q1118A(NBD2) suggested that the P-gp transport mechanism shows redundancy (47). It was found that the single Q475A or Q1118A mutants retained transport activity and 35–50% of wild-type ATPase activity but the double Q475A/Q1118A mutant was inactive. These results with the Q-loop mutations suggest that ATP hydrolysis at one site might be sufficient for transport (47). A similar mechanism might operate in other ABC proteins (48, 49).

**FIGURE 6.**
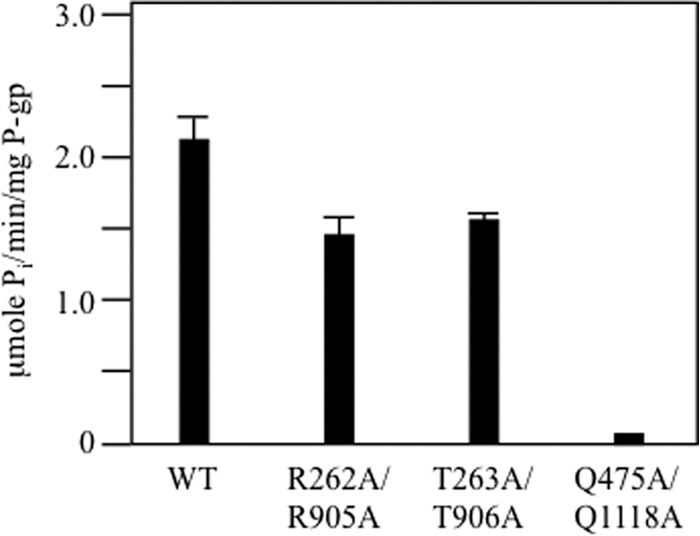
**Double mutations introduced into equivalent positions in IH2 and IH4 caused modest reductions in ATPase activity.** Histidine-tagged wild-type P-gp (WT) or mutants containing point mutations to equivalent positions in IH2 and IH4 (R262A(IH2)/R905A(IH4), T263A(IH2)/T906A(IH4)), or NBD1 and NBD2 (Q475A(NBD1)/Q1118A(NBD2)) were expressed in HEK 293 cells, isolated by nickel-chelate chromatography and assayed for ATPase activity in the presence of verapamil. The results are derived from the mean of three different transfections + S.D.

We constructed histidine-tagged R262A/R905A and T263A/T906A mutants (in wild-type background) to test if mutations in both IH2 and IH4 were needed to inactivate P-gp ATPase activity. It was found that both mutants retained about 70% of wild-type verapamil stimulated ATPase activity (Fig. 6). A Q475A/Q1118A control P-gp however, showed little detectable ATPase activity. The results show that residues Arg-262 or Arg-905 were not essential for coupling of drug binding to activation of ATPase activity.

## Discussion

**FIGURE 7.**
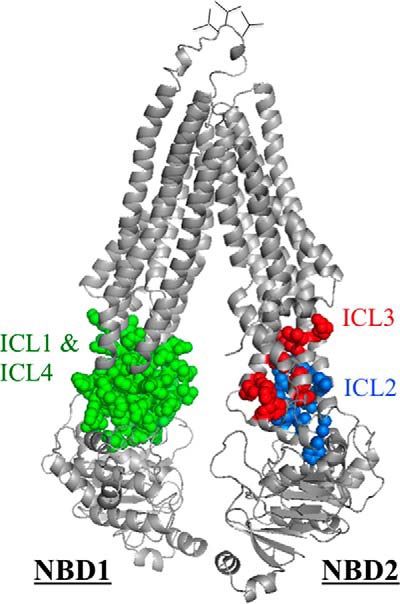
**P-gp maturation is highly sensitive to point mutations at the second transmission interface but relatively insensitive to changes at the first transmission interface.** Predicted structure of human P-gp in an open conformation (based on the crystal structure of mouse P-gp) (56). Mutation of residues in ICL2 and ICL3 inhibited maturation of P-gp. The side chains of these residues are shown in *blue* and *red*, respectively. None of the mutations in ICLs 1 and 4 inhibited maturation of P-gp and their side chains are shown in *green*.

The major novel mechanistic findings in this study were that the transmission interfaces make asymmetric contributions to folding and activity of P-gp. The results show that the NBD2 transmission interface is particularly important for folding of P-gp and helps to explain why P-gp maturation is sensitive to deletion of NBD2 (26). The NBD2 transmission interface appeared to require formation of a very precise tetrahelix bundle structure between ICL2 and ICL3 because P-gp maturation was highly sensitive to changes in these segments (Fig. 7). About half the ICL2 point mutations and a third of the ICL3 point mutations blocked maturation (Fig. 2, *B* and *D*).

By contrast, maturation of P-gp was highly insensitive to changes at the NBD1 transmission as none of the ICL1 or ICL4 point mutations severely reduced maturation (Fig. 7). Apparently, P-gp only required a hydrophilic flexible NBD1 transmission interface as maturation was relatively insensitive to structural perturbations of the ICL1/ICL4 tetrahelix bundle or IH1/4 segments.

The NBD2 contact site required more precise and hydrophobic interactions for P-gp maturation and activity compared with NBD1. The F1086R or Y1087R mutations at the NBD2 socket blocked maturation and activity while P-gp showed substantial activity when the comparable mutations (L443R, Y444R) were made to the NBD1 socket.

Indeed, a very conservative change of Y1087F in NBD2 reduced P-gp activity by about 70% (Fig. 4*B*). Removal of the hydroxyl side chain may have detrimental effects on activity because the equivalent residue in the crystal structure of *C. elegans* P-gp (Tyr-1129) shows hydrogen bond interactions with Asp-846 in IH3 (equivalent to human Asp-805) and Arg-286 in IH2 (equivalent to human Arg-262) (21). In a modeling study of human P-gp, it was also predicted that the hydroxyl group of Tyr-1087 would form a hydrogen bond with Asp-805 (24). All other replacements to Tyr-1087 (Ala, Leu, Arg, and Glu) reduced P-gp maturation and activity to undetectable levels.

The *C. elegans* P-gp structure showed that the equivalent residues at the NBD1 transmission interface made the same contacts (21). The tyrosine at position 468 in NBD1 (equivalent to human Tyr-444) formed hydrogen bonds with Asp-188 in IH1 (equivalent to human Asp-164) and Arg-946 (equivalent to human Arg-905). In the human P-gp modeling study, it was also predicted that the hydroxyl group of Tyr-444 would form a hydrogen bond with Asp-164 (24). The presence of a hydroxyl group at position 444 was not essential however, as the Y444F mutant yielded wild-type levels of maturation and activity (Fig. 4).

Differences between equivalent IH4-NBD1 and IH2-NBD2 contact sites were also observed in a cross-linking study (7). Both mutants L443(NBD1)/S909C(IH4) and A266C(IH2)/F1086C(NBD2) could be cross-linked with copper phenanthroline but only the L443C/S909C mutant was active. The activity of this mutant was lost upon cross-linking and restored with dithiothreitol.

Residues Asp-164 in IH1 and Asp-805 in IH3, predicted to form hydrogen bonds with Tyr-444 and Tyr-1087 respectively, have also been postulated to interact with the adenine ring of ATP (50). To test if residues Asp-164 or Asp-805 were essential for activity, Kapoor *et al.* (51) tested the effects of introducing D164C or D805C mutations into human Cys-less P-gp. They found that both mutations reduced trafficking of P-gp to the cell surface when they were expressed in HeLa cells. The D164C mutation reduced cell surface expression to about 40% of the Cys-less parent while the presence of both the D164C and D805C mutations reduced cell surface expression to about 20% of the parent. Immunoblot analysis of whole cell extracts expressing D164C/D805C showed that the major product was immature P-gp. The observation that P-gp mutants R262A/R905A, T263A/T906A (this study), and D164C/D805C (51) retain substantial activity suggests that there are multiple NBD-TMD contacts as reported by Jin *et al.* (21).

The D164C/D805C mutant could be efficiently rescued when expressed in the presence of cyclosporine A to yield mature P-gp as the major product. The rescued mutant showed transport levels of various substrates (rhodamine 123, NBD-cyclosporine, daunomycin, calcein-AM) similar to the Cys-less parent. Their results showed that Asp-164 or Asp-805 were not critical for drug transport. In agreement with cysteine mutagenesis results, we found that the D164A mutation did not reduce verapamil-stimulated ATPase activity (23).

A difference was that the D164A mutation did not reduce maturation of P-gp (23). The likely explanation is that the D164C mutation was introduced into a Cys-less P-gp background while the D164A mutation was introduced into a wild-type background. Mutation of P-gp's 7 endogenous cysteines reduces its maturation efficiency to make the protein more sensitive to processing mutations. For example, the F1086C mutation inhibits maturation of Cys-less P-gp but not wild-type P-gp (23).

Our results suggest that the NBD2 transmission interface is particularly sensitive to mutations relative to the NBD1 transmission interface. Studies on mutants of P-gp's structurally similar sister proteins, ABCB4 and CFTR, also suggest that the NBD2 transmission interface plays a key role in activity and protein assembly.

Human ABCB4 is a phosphatidylcholine transporter that shows 78% amino acid identity to P-gp (52). An ABCB4 Q1174E mutant was predicted to be the cause of progressive familial intraheaptic cholestasis type 3 in a patient (53). Analysis of the mutant suggested that the mutation disrupted the NBD2 transmission interface to inhibit substrate transport and substrate-induced ATPase activity (53).

CFTR is a chloride channel predicted to be structurally similar to P-gp (25). The NBD2 transmission interface was also found to be particularly important for CFTR assembly as processing mutations in other domains that cause cystic fibrosis (such as ΔF508 in NBD1, G91R in TMD1, L1093P in TMD2) were found to impair the conformational stability of NBD2 (54). Defects in folding of NBD2 can also be relayed through the second transmission interface to impair folding of the rest of the protein. For example the CFTR N1303K (54) and P-gp L1260A (55) NBD2 mutations inhibit maturation of the proteins.

NBD2 is more important for P-gp maturation than CFTR. A ΔNBD2 P-gp truncation mutant did not mature while ΔNBD2 CFTR showed robust maturation (26). The results of the present study suggest that NBD2 interactions are critical for stabilizing the ICL2/ICL3 tetrahelix bundle. The sensitivity of the P-gp second transmission interface to point mutations makes it an attractive target to develop compounds to inhibit activity or delivery of the protein to the cell surface to increase the effectiveness of chemotherapy or enhance drug delivery.

All of the ICL2/ICL3 processing mutations could be repaired with tariquidar. Binding of tariquidar to the TMDs (41) might stabilize the ICL2/ICL3 interactions in these mutants and promote proper interactions between various domains. The implication of these results is that binding of compounds to the TMDs of processing mutants in other ABC proteins could be a potential method for overcoming their folding defects.

## Footnotes

2 The abbreviations used are:

ABC: ATP-binding cassette
P-gp: P-glycoprotein
NBD: nucleotide-binding domain
HEK: human embryonic kidney
TM: transmembrane
TMD: transmembrane domain
ICL: intracellular loop
IH: intracellular helix.
